# Case report: Hand-arm vibration syndrome in a dental technician

**DOI:** 10.3389/fpubh.2024.1424236

**Published:** 2024-10-01

**Authors:** Jonathan Wen Yu Lam, Yew-Long Lo, Yuke Tien Fong

**Affiliations:** ^1^Department of Radiology, Tan Tock Seng Hospital, Singapore, Singapore; ^2^Department of Neurology, National Neuroscience Institute, Singapore, Singapore; ^3^Department of Occupational and Environmental Medicine, Singapore General Hospital, Singapore, Singapore

**Keywords:** vibration, hand-arm vibration (HAV), dental, hand tools, repetitive strain injury

## Abstract

Occupational exposure to vibration using hand-held tools may cause hand-arm vibration syndrome (HAVS) among workers. We report the case of a 39-year-old lady with a 5-year work exposure to repetitive wrist movements and vibration from hand tools in the dental fabrication laboratory, working as a dental technician. She presented with a 3-year history of chronic pain over the wrists and positive symptoms of numbness and tingling in the hands, pain and discomfort of the fingers during cold exposure, and poor grip strength. Symptoms over the right hand were worse than the left. She is right-hand dominant. She had bilateral median nerve neuropathy at the wrist. Nerve conductive tests were consistent with minimal bilateral carpal tunnel syndrome. MRI showed evidence of soft tissue damage from repetitive strain injury of the right wrist. Neurosensory grading for hand-vibration syndrome (HAVS) using the Stockholm Workshop Scale (SWS) was performed and she was graded as Stage 1SN with numbness or tingling symptoms. Using the International Consensus Criteria (ICC) grading for HAVS, she was graded Stage N1 with numbness and/or tingling (symptoms) of finger. A workplace risk health assessment (WHRA) was performed, and exposure scores for her work tasks in dental fabrication and risk levels were determined using the HSE (Health and Safety UK) Assessment of Repetitive Tasks (ART) tool. The processes of teeth setting using dental burs (exposure score 23), divestment work with stone clippers (score 20), and use of pneumatic drills (score 21) were deemed high-risk activities for repetitive strain injury. The use of carving tools (score 12), packing with the use of flask clamps, and trimming (score 14) were classified as medium high-risk activities for repetitive strain injuries. Workplace modifications and workplace vibration exposure level monitoring protocols were subsequently established with the stabilization of the patient's symptoms.

## Case presentation

We report the case of a dental technician who was exposed to both repetitive movements and vibration tools at work. Ms X, who is 39 years old, has worked as a dental technician for 5 years in a dental fabrication laboratory. She developed bilateral carpal tunnel syndrome and repetitive strain injury involving the right wrist. Workplace evaluation was performed to delineate the work risks in her daily routine at the laboratory.

The Health and Safety Executive (HSE) Assessment for Repetitive Tasks (ART) (1) tool was utilized to evaluate the risks of exposure to repetitive strain injuries.

She complained of bilateral chronic wrist pain, worse over the right wrist, for the preceding 3 years, and the discomfort waxed and waned periodically, fluctuating with her workload. She also noted some tingling sensation and cramping in the right hand. She was referred for further evaluation for suspected work-related injury. There were no other joint pains and no history of other trauma or rheumatological disease. She is right- hand dominant. The case was reviewed for the possibility of hand-arm vibration syndrome in view of the clinical picture, setting, and work exposure to vibration with hand tools in the dental laboratory.

Occupational history was reviewed using the HSE Health Surveillance Guide for Occupational Health Professionals (2). The patient had significant exposure to repetitive movements and the use of vibration tools in her work, particularly during dental fabrication. Assessment of symptoms was made following the format in the UK HSE guidelines (1). The patient indicated positive symptoms of numbness or tingling, also occurring at night, discomfort in the fingers and hands during work and cold exposure, and poor hand grip strength.

Clinical evaluation showed slight tenderness over the right lunate. There was no wasting of the hand muscles and capillary return time was normal. A preliminary assessment using the Camry Digital Handgrip Dynometer for hand grip strength was made at the outpatient clinic. This instrument is validated as being equivalent to the Jarmal Dynometer, which is the gold standard for measuring hand grip (3). This was diminished with results showing reduced right- hand grip with a dynamometer reading of 18 kg/F (age and gender-matched values-−30.8 kg/F, 58.4% of normal), and 14 kg/F (normal 25.8 kg/F, 54.2% of normal) on the left hand.

Formal functional evaluation of the hands and wrists with sensory and motor mapping was performed. Sensation testing was performed using the 2-point discrimination test. These were within normal limits at 3–4 mm for all the fingers of both hands.

On motor testing, the range of movements of the wrists showed reduced active flexion on the right wrist at 72° and left wrist 80° (*N* = 90); Active extension was normal at 74° at the right wrist and 76° for the left (*N* = 70–90). Thumb opposition was normal at (R): 10/10 and (L): 10/10 for the Kapandji thumb score.

The JAMAR dynamometer was used to test hand-grip strength for formal motor testing. The results are consistent with the preliminary assessment. Hand grip strength tested for the right hand at Jamar's position was reduced at 11.6 kg/F (57% of normal) as well as the left, measuring 10.6 kg/F (52.2% normal for her age and gender).

Pinch Strength was tested for the fingers and was found to be reduced. The average lateral pinch strength was 2.3 kg/F (51.1% of normal) for the right hand and 3.1 kg/F (68.9% of normal) for the left hand. The results of this evaluation are available in Supplementary material.

Neurosensory grading using the Stockholm Workshop Scale (SWS) for HAVS was Stage 1SN with symptoms of numbness or tingling symptoms (intermittent numbness with or without tingling) – (full grading system in Supplementary Table 1).

Grading using the International Consensus Criteria (ICC) was Stage N1 with numbness and/or tingling symptoms (intermittent numbness and/or tingling of fingers)—Supplementary Table 2.

### Occupational history

This was reviewed using the HSE Health Surveillance Guide for Occupational Health Professionals (2). The patient had significant exposure to repetitive movements and the use of vibration tools in her work, particularly during the dental fabrication processes.

**Figure 1:**
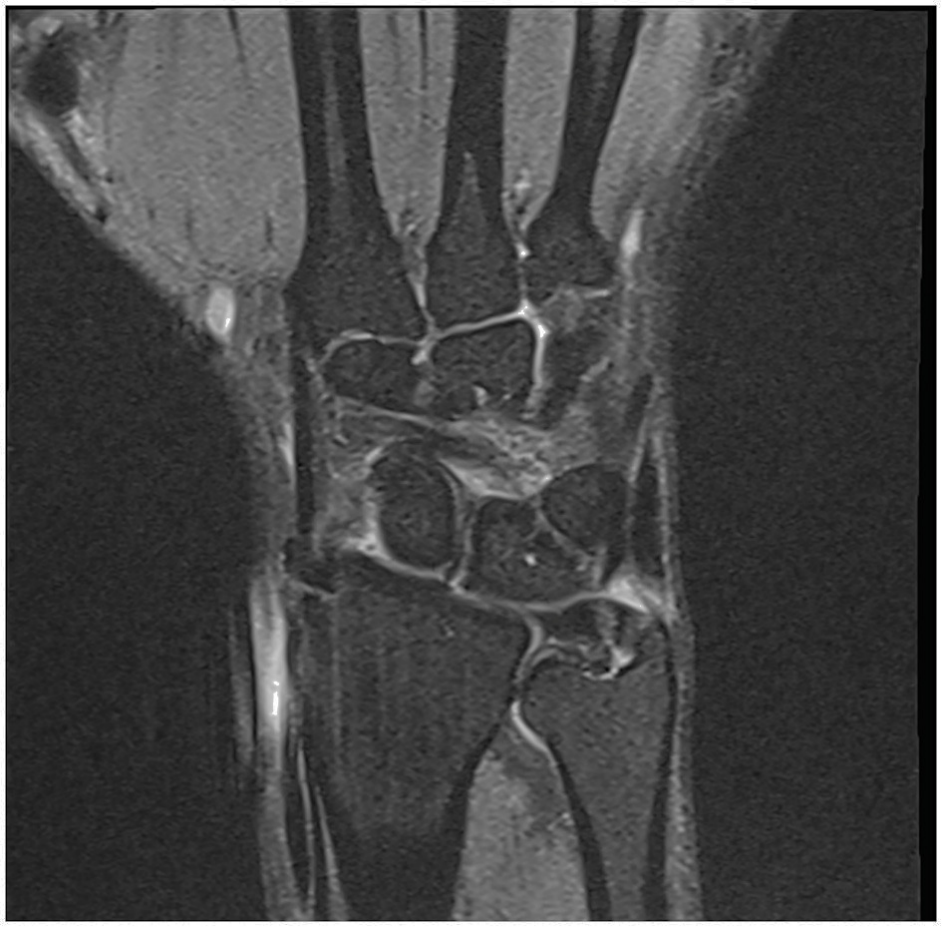
Coronal MRI of the right wrist demonstrating a tear in the triangular fibrocartilage.

Investigations performed MRI of the right wrist showed evidence of wrist injury with partial tear of the triangular fibrocartilage complex (Figure 1), partial tear of the scapholunate ligament and mild extensor carpi ulnaris tenosynovitis.

An MR angiogram of the vessels of the right hand was normal.

We referred the patient to a neurologist who performed nerve conduction studies. The Nerve Conduction Studies (NCS) were suggestive of median neuropathy at both wrists. There was no electrophysiological evidence of sensorimotor polyneuropathy. Motor nerve conduction study (NCS) results for the lumbrical and interosseous muscles showed delayed latency for the median nerve compared to the ulnar nerves. The sensory nerve conduction studies for the median and ulnar nerves showed normal latency, amplitude, and conduction velocity for both the distal and proximal segments. The median-ulnar interossei comparison study was performed (0.59 millisecond on the right; 0.73 milli second on the left; normal < 0.5 ms). The results of the NCS have been uploaded into the Supplementary material. Based on the NCS results, the patient was graded as having minimal median neuropathy at both wrists (4, 5).

Evaluation of the main hand vibrating tool (6) was performed. It has a torque of up to 7 Newton cm (Ncm) and a speed of 1,000–50,000 rpm (clockwise operation). Counterclockwise rotation was limited to 5,000 rpm. It has a soft grip surface for comfortable handling and a secure grip. The device measured 165 mm long with a diameter range of 25–29 mm (oval for peeling grip). The handpiece pen grip diameter was 14 mm with a weight of 250 g. The manufacturer's specifications of the laboratory handpiece indicated that it is reliable, effective, and suitable for daily work in dental laboratories.

In a study by Kaulbars (7), the authors commented that in dental laboratories, employees mainly use straight grinders with chucks when processing carriers for dental prostheses. Dental technicians spend a large part of their working day using this equipment. In addition to vibration from the grinder, vibration is also transmitted by the handheld workpiece—a risk that has so far been disregarded in the manufacturer's information. This paper examined the system of vibration measurement in different working conditions and work processes with the special problems of conducting measurements on small workpieces. The findings provide a basis for risk analysis and for the design of prevention measures.

## Work process evaluations for repetitive movements from workplace health risk assessment

This evaluation was performed to understand the contributing factors to the injury from vibration and repetitive movements of the upper limbs.

### Job scope and job processes

She had been in her current job for 5 years but she was symptomatic for 3 years. Her job processes included three key areas of which the major component was in denture fabrication. The denture fabrication workload was performed at an average of 5 units per week, 9 units in 2 weeks. A unit of the work refers to a process whereby the dental technician completes the tasks of teeth setting, wax manipulation, investment and packing functions, divestment work using tools like stone clippers, pneumatic drills, and gross and fine trimming and polishing. These processes are included in the fabricating of full or partial dentures. The work involved in the repair of dentures was not included in the workload described.

The key responsibilities of the dental technician are detailed in Supplementary Table 3.

A description of the key work processes in dental fabrication is shown in Supplementary Table 4 and a summary of the findings of the WHRA using the ART tool to evaluate repetitive movements is shown in Table 1.

**Table 1:** Summary of work processes and the ART tool (1) evaluation results.

	**Work processes**	**Task score**		**Exposure score**		**Risk level**	
		**Left** ^*^	**Right** ^**^	**Left** ^*^	**Right** ^**^	**Left** ^*^	**Right** ^**^
1	**Teeth setting, wax manipulation**						
a	Use of carving tools.	8	12	8	12	Low	Medium
b	Use of dental burs.	15	23	15	23	Medium	High
2	**Investment, packing**						
	Packing and using flask clamp	4	13	4	13	Low	Medium
3	**Divestment work using tools**						
a	Using stone clippers.	11	20	11	20	Low	High
b.	Using pneumatic drills.	14	21	14	21	Medium	High
4	**Gross and fine trimming and polishing**						
	Trimming and polishing	14	14	14	14	Medium	Medium

^*^Left upper limb.

^**^Right upper limb.

The task score is the summary score of the activity as calculated from the HSE UK ART tool (1).

The exposure score in the HSE UK ART tool is calculated from the task score and duration multiplier using the equation Exposure score = Task score × Duration multiplier.

The processes of teeth setting using dental burs (exposure score 23), divestment work with stone clippers (score 20), and use of pneumatic drills (score 21) are high-risk activities for repetitive strain injury. Use of carving tools (12), packing with the use of a flask clamp, and trimming (14) are medium high-risk activities.

Using burs, pneumatic drills, trimming and polishing have risks of vibration exposure. The use of clippers in stone clipping involves the use of significant force, which can damage the triangular fibrocartilage complex (TFCC) and compress the neurovascular structures at the wrist.

Based on the assessment (Table 2), this provided the impetus for Ms. X's employer to institute workplace modifications and establish workplace vibration surveillance and safety protocols, with subsequent stabilization of Ms. X's symptoms.

**Table 2:** Risk stratification and recommendations for the ART tool (1).

**Exposure score**	**Risk level**	**Risk color coding**	**Recommendations**
0–11	Low	Green	Consider individual circumstances
12–21	Medium	Orange	Further investigation and work modifications required
22 or more	High	Red	Further investigation and work modifications required urgently

### Workplace modifications and improvements

The following workplace modifications were made by the organization where the patient worked in response to this case.

The management checked for and replaced faulty instruments and made a commitment to reinforce regular maintenance schedule for the laboratory handpieces. They identified high-risk processes in the workplace for sources of vibration and implemented administrative measures to reduce the exposure time to vibration for high-risk workers and high-risk processes. These included streamlining the use of equipment with vibration issues to minimize exposure, monitoring the time of exposure to vibration and keeping an exposure time log for each worker exposed to vibration.

Mandatory regular breaks and task rotations were introduced. Risk assessments for early identification of cases were introduced. Screening questionnaires were introduced to monitor staff for symptoms regularly. Annual medical checkups with occupational physicians were scheduled for staff with significant work exposure to vibration tools.

An ergonomic evaluation was made at the work site to identify and remediate ergonomic issues that could precipitate repetitive musculoskeletal problems. Staff were taught to keep optimal working postures. Educational talks were conducted, and related educational materials were provided to educate staff on the risks of vibration and repetitive strain injury.

A plan was made to progressively eliminate or reduce tasks that involve prolonged or intense vibration. For example, in, the laboratory, heavy-duty trimming was in the process of being phased out. The production and trimming of metal frameworks with higher vibration risks were also outsourced.

The patient was exempted from work with vibration to allow for her recovery. The patient's symptoms stabilized after a month. She subsequently made a personal decision to change her vocation.

## Discussion

Occupational diseases are preventable. The early identification of risk factors and risk stratification of tasks is a critical initial step in protecting the health of workers exposed to work risks and preventing injury and disease onset. Prevention and control of excessive and prolonged vibration transmission to the hands at work is the most important step in preventing vibration injury.

Many clinical and epidemiological studies have shown an increased risk of upper limb disorders due to biomechanical overload, functional stress (typically, repetitive movements), and repeated microtrauma caused by hand tool vibrations (8, 9). This set of injuries, involving anatomical and functional structures of the upper limb, is defined as the hand-arm vibration syndrome. This is a disease classification characterized by a vascular component, a neurological component, and an osteoarthritic component (10). The vascular component is represented by a secondary form of Raynaud's phenomenon, Vibration-induced White Finger (VWF).

The neurological component consists of predominantly sensory peripheral neuropathy with multifocal distribution, or it may be one confined to the fingers. There is finger paresthesia, reduced tactile and thermal sensitivity, limited manual dexterity, and decreased ability in fine manipulation. Our patient had bilateral minimal carpal tunnel syndrome with symptoms and findings consistent with HAV.

Dental technicians are exposed to multiple hazards at work (11). Apart from chemical and physical hazards such as noise and dust, dental technicians are also exposed to hand/arm vibrations while working with various appliances and tools. Long-term exposure may result in the “white finger syndrome”.

Cherniack et al. (12) performed a cross-sectional study of 94 experienced dental hygienists, investigating the peripheral nerve function and clinical signs and symptoms of HAV. The study concluded that the high incidence of paresthesia observed among dental hygienists appears to be attributable to several pathophysiological mechanisms, including sensory nerve demyelination at the carpal tunnel and intrinsic to the digits and dysfunction of fingertip mechanoreceptors. A distinct sub-population appears to exhibit a high level of accumulated abnormality.

In the European Union, vibration is regulated under the Directive 2002/44/EC (13) on the minimum health and safety requirements regarding workers' exposure to the risks arising from physical agents (vibration).

This directive aims to protect workers from the risks associated with exposure to vibration, including HAVS and whole-body vibration (WBV). The directive establishes exposure limit values for hand-arm vibration and whole-body vibration. These values are based on the daily and weekly exposure levels. The requirements cover employer responsibilities, control measures, health surveillance requirements, training and information on how to use tools and machinery safely, record-keeping of risk assessments, vibration measurements, control measures implemented, and health surveillance results.

## Conclusion

This case illustrates the importance of early detection and the need to implement surveillance for high-risk healthcare workers exposed to vibration work to manage risks. Similar regulations as in the European Union should be established to protect dental workers at risk for hand-arm vibration syndrome.

## Data Availability

The original contributions presented in the study are included in the article/Supplementary material, further inquiries can be directed to the corresponding author.
