# Supplementary material for: Case report: Hand-arm vibration syndrome in a dental technician
Source: Front Public Health. 2024 Oct 1;12:1424236. doi: 10.3389/fpubh.2024.1424236 (PMC11473303; doi:10.3389/fpubh.2024.1424236)
Supplement: Supplementary file 1 [file Data_Sheet_1.docx]

**Supplementary materials**

| **Table 1 Grading of neurosensory manifestations using the Stockholm Workshop Scale (1)[3]** |
| --- |
| Stage 0SN: no numbness or tingling (vibration exposed but no symptoms). |
| Stage 1SN: numbness or tingling symptoms (intermittent numbness with or without tingling). |
| Stage 2SN: as in 1SN together with impaired perception of touch, vibration, temperature and/or pinprick test (findings)(intermittent or persistent numbness, reduced sensory perception). |
| Stage 3SN: as in 2SN together with impaired tactile discrimination (2PD) or manipulative dexterity (Purdue Pegboard) (intermittent or persistent numbness, reduced tactile discrimination and/or manipulative dexterity). |

| - **Table 2 Severity of HAVS grading according to the International Consensus Criteria [ICC].[4]** |
| --- |
| - Stage N0: exposed to vibration but no numbness or tingling (no symptoms). |
| - Stage N1: numbness and/or tingling (symptoms) (intermittent numbness and/or tingling of fingers). |
| - Stage N2: as in N1 and impairment in two out of three sensory modalities; perception of touch, vibration or temperature (findings) (as in stage N1 but with sensory perception loss as evidenced by two or more validated methods such as monofilaments, thermal esthesiometry or vibrotactile thresholds). |

Table 3 Key responsibilities of a dental technician

| Restoring or replacing natural teeth by fabricating crowns, fixed and removable bridges, dentures, and dental implants; |
| --- |
| constructing dental prostheses for implantology and maxillo-facial reconstructions; receiving physical dental impression moulds or computerized models from dental practices and studying dentists' instructions; |
| encasing wax replicas of dental impressions with suitable mould materials; |
| constructing prostheses and devices by replacing wax with plastic, metal, or ceramic materials; polishing and finishing dental prostheses and devices prior to placement by dentists; |
| making adjustments to dental prostheses and devices before final placement; |
| managing supply inventories, performing routine equipment maintenance and facilitating repairs; documenting processes, as well as adhering to industry regulations and safety standards; |
| keeping informed of advancements in the field of dental laboratory technologies and techniques. |

**Table 4 : Key processes in dental fabrication**

Dentists send the technicians a specification of the item (crown, bridge, prosthesis) to be made, along with an impression (mold) of the patient’s mouth or teeth. Dental technicians then create a model of the patient’s mouth by pouring plaster into the impression and allowing it to set. The next step is to place the model on an apparatus that mimics the bite and movement of the patient’s jaw. The model serves as the basis of the prosthetic device. Technicians examine the model, and based on these observations and the dentist’s specifications, build and shape a wax model, using small hand instruments called wax carvers. They use this wax model to cast the metal framework for the missing tooth or teeth. Dental technicians then prepare the metal surface to allow the alloy and porcelain to bond, by using small handheld tools and sand-blasting. They then apply porcelain in layers, to achieve the desired shape and color of a tooth. Technicians then place the tooth in a porcelain furnace to bake the porcelain onto the metal framework, and then adjust the shape and color, with subsequent grinding and addition of porcelain to achieve a sealed finish. The final product is a nearly exact replica of the lost tooth or teeth.

| 1. **Teeth setting, wax manipulation** |
| --- |
| Teeth setting refers to the precise arrangement of artificial teeth in a denture base (either complete or partial). |
| Wax manipulation involves shaping and molding dental wax for various purposes in dentistry. Carving tools are used to sculpt the wax to the desired form. Dental burs are used in trimming and finishing processes to remove excess wax and refine the contours, and polishing smooths the wax surface for aesthetics and comfort. |
| 1. **Investment and packing** |
| **Investment** refers to the material used to create a mold (also known as a flask) for casting dental restorations (such crowns, bridges, dentures, etc.). The p**urpose is t**o create an accurate negative mold of the wax pattern (created from the patient’s oral impression. The investment material forms the mold cavity for casting the final dental restoration. The steps include |
| - Wax Try-In: The wax denture base (try-in) is placed in the patient’s mouth to verify fit, aesthetics, and occlusion. Any adjustments are made to the wax try-in. |
| - Packing Process: The processed acrylic resin (usually heat-cured) is packed into the mould using a flask. The flask consists of two halves: the base and the lid. The acrylic resin is mixed and placed in the base of the flask. The wax try-in is positioned in the flask, and the lid is on top. The flask is heated to cure the acrylic resin. |
| - Finishing and Polishing: The processed denture is removed from the flask. Excess material is trimmed and polished to achieve the final shape and surface texture. |
| 1. **Divestment work using tools**. |
| This refers to the process of removing the investment material (usually a refractory material) from a cast restoration after it has been cast. It is a critical step in obtaining the final dental restoration. |
| After casting, the flask is heated to remove the wax pattern (burnout). The investment material disintegrates, leaving the metal framework. The cast restoration is carefully removed from the investment mold. The metal framework is inspected, cleaned, and prepared for further processing. |
| Stone clippers are used for trimming the cast, removing excess material and creating smooth margins: |
| The cast restoration is carefully removed from the investment mold. Stone clippers are used to trim off any investment material that adheres to the metal framework. The margins are refined to achieve the desired fit and finish. Pneumatic drills are commonly used in dental procedures, but not specifically for divestment. |
| 1. **Gross and fine trimming and polishing** |
| Trimming involves removing this excess material to achieve the desired fit and contour. After casting a dental restoration (such as a crown, bridge, or denture), excess material (usually acrylic or metal) remains around the edges. Polishing enhances the aesthetics, comfort, and longevity of dental restorations. |
